# Decline in forced vital capacity in subjects with systemic sclerosis-associated interstitial lung disease in the SENSCIS trial compared with healthy reference subjects

**DOI:** 10.1186/s12931-022-02095-6

**Published:** 2022-07-05

**Authors:** Toby M. Maher, Arnaud Bourdin, Elizabeth R. Volkmann, Serena Vettori, Jörg H. W. Distler, Margarida Alves, Christian Stock, Oliver Distler

**Affiliations:** 1 Keck School of Medicine, University of Southern California, Los Angeles, CA USA; 2 National Heart and Lung Institute, Imperial College, London, UK; 3 PhyMedExp, University of Montpellier, INSERM U1046, CNRS, UMR 9214, Montpellier, France; 4 Department of Respiratory Diseases, University of Montpellier, CHU Montpellier, Montpellier, France; 5 Department of Medicine, Division of Rheumatology, David Geffen School of Medicine, University of California, Los Angeles, CA USA; 6 UOC Di Fisiopatologia E Riabilitazione Respiratoria, Ospedale Monaldi, Naples, Italy; 7 Department of Internal Medicine 3-Rheumatology and Immunology, Friedrich-Alexander-University Erlangen-Nürnberg (FAU), Universitätsklinikum Erlangen, Erlangen, Germany; 8 TA Inflammation Med, Boehringer Ingelheim International GmbH, Ingelheim, Germany; 9 Global Biostatistics and Data Sciences, Boehringer Ingelheim Pharma GmbH & Co. KG, Ingelheim Am Rhein, Germany; 10 Department of Rheumatology, University Hospital Zurich, University of Zurich, Zurich, Switzerland

**Keywords:** Connective tissue diseases, Pulmonary fibrosis, Scleroderma, systemic, Vital capacity

## Abstract

**Background:**

The forced vital capacity (FVC) of healthy individuals depends on their age, sex, ethnicity and height. Systemic sclerosis-associated interstitial lung disease (SSc-ILD) is characterised by loss of FVC. We compared FVC values in the subjects with SSc-ILD in the SENSCIS trial of nintedanib versus placebo with values from hypothetical matched healthy references.

**Methods:**

The SENSCIS trial enrolled subjects with SSc with first non-Raynaud symptom in the prior ≤ 7 years, extent of fibrotic ILD on HRCT ≥ 10%, and FVC ≥ 40% predicted. FVC at baseline and decline in FVC over 52 weeks were compared with FVC values in hypothetical healthy reference subjects matched 1:1 to the subjects in the trial for age, sex, ethnicity and height, determined using equations published by the European Respiratory Society Global Lung Function Initiative.

**Results:**

At baseline, mean (SD) FVC was 2460 (737) mL in the nintedanib group (n = 287) compared with 3403 (787) mL in the hypothetical matched healthy references. Mean (SD) FVC was 2544 (817) mL in the placebo group (n = 286) compared with 3516 (887) mL in the hypothetical matched healthy references. Mean (SE) changes in FVC at week 52, i.e., age-related loss of lung function, in the hypothetical healthy references matched to the nintedanib and placebo groups, respectively, were − 26.3 (0.5) mL and − 25.8 (0.5) mL. The difference in the change in FVC at week 52 between the nintedanib group and the hypothetical healthy references was 26.6 mL (95% CI: 1.2, 52.0; p = 0.04). The difference in the change in FVC at week 52 between the placebo group and the hypothetical healthy references was 77.5 mL (95% CI: 51.4, 103.7; p < 0.0001).

**Conclusions:**

Subjects with SSc-ILD in the SENSCIS trial had impaired lung function at baseline and experienced further deterioration over 52 weeks. The decline in FVC in the placebo group was four-fold greater than in a hypothetical group of matched healthy references, whereas the decline in FVC in patients who received nintedanib was two-fold greater than in hypothetical healthy references. These data highlight the clinical relevance of the slowing of FVC decline provided by nintedanib.

*Trial registration* Registered 5 November 2015, https://clinicaltrials.gov/ct2/show/NCT02597933.

**Supplementary Information:**

The online version contains supplementary material available at 10.1186/s12931-022-02095-6.

## Background

Interstitial lung disease (ILD) is a common manifestation of systemic sclerosis (SSc) [1] and is the leading cause of death in patients with SSc [2]. SSc-ILD is typically associated with impairment in forced vital capacity (FVC) although FVC is preserved in some patients [1, 3]. The greatest loss of lung function tends to occur early in the course of SSc [4–6], but the natural history of SSc-ILD is variable [3, 7]. At any level of FVC, in the absence of another cause, decline in FVC reflects the progression of SSc-ILD and is associated with an increased risk of hospitalization and mortality [1, 8–10].

Healthy individuals have varied FVC depending on their age, sex, ethnicity and height. FVC declines gradually with aging. The FVC that a healthy individual is expected to have can be determined using reference equations [11–13], but a single value for FVC % predicted may be less informative than looking at changes over time.

Nintedanib is an intracellular inhibitor of tyrosine kinases that inhibits processes fundamental to the progression of fibrosis [14]. In the SENSCIS trial, nintedanib was estimated to reduce the rate of decline in FVC (mL/year) over 52 weeks by 44% compared with placebo, with no heterogeneity in its treatment effect detected across subgroups based on sex, age (< 65 vs ≥ 65 years), race, anti-topoisomerase I antibody (ATA) status, limited cutaneous versus diffuse cutaneous disease, or respiratory symptoms [15–17]. Further, a smaller proportion of subjects treated with nintedanib than placebo had an absolute decline in FVC of > 5% to ≤ 10% predicted (13.6% vs 20.1%) or > 10% to ≤ 15% predicted (3.5% vs 5.2%) over 52 weeks [18].

To obtain insights into the decline in lung function in patients with SSc-ILD, and to put the findings of the SENSCIS trial into context, we compared FVC at baseline and the decline in FVC over 52 weeks in the SENSCIS trial with values in hypothetical healthy reference subjects matched to the SENSCIS trial subjects for age, sex, ethnicity and height. We also estimated the “effective lung age” of subjects in the SENSCIS trial (i.e., the age of healthy individuals with the same FVC) and compared these estimates to their real age.

## Methods

### Trial design

The design of the SENSCIS trial (NCT02597933) has been described and the protocol is publicly available [15]. Briefly, subjects had SSc with onset of first non-Raynaud symptom in the prior ≤ 7 years, an extent of fibrotic ILD on HRCT ≥ 10%, FVC ≥ 40% predicted and diffusion capacity of the lung for carbon monoxide (DLco) 30–89% predicted. Subjects taking prednisone ≤ 10 mg/day and/or stable therapy with mycophenolate or methotrexate for ≥ 6 months were allowed to participate. Subjects were randomised to receive nintedanib 150 mg twice daily (bid) or placebo, stratified by ATA status. Subjects remained on blinded treatment until the last subject had reached week 52 but for ≤ 100 weeks.

### Analyses

**Fig. 1:**
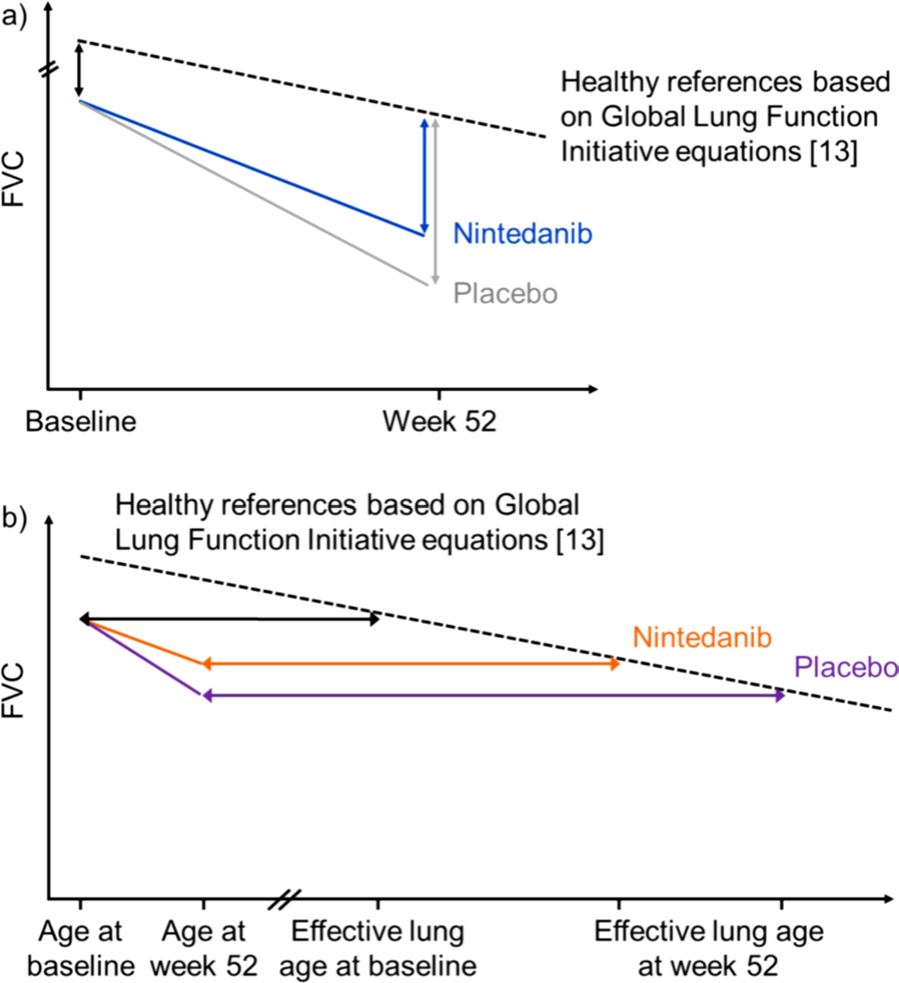
Schematic illustration of **a** the course of FVC decline in patients in the SENSCIS trial and healthy reference subjects and **b** the difference between effective lung age and actual age in subjects in the SENSCIS trial. In figure a, the black dashed line denotes the decline in FVC in the hypothetical healthy reference population; the coloured lines denote the decline in FVC in the nintedanib (blue) and placebo (grey) groups in the SENSCIS trial; the coloured vertical arrows denote the differences in FVC between the nintedanib (blue) and placebo (grey) groups in the SENSCIS trial and the hypothetical healthy reference population at week 52. In figure b, the black horizontal line denotes the difference between actual age and effective lung age at baseline; the coloured horizontal arrows denote the differences between actual age and effective lung age at week 52 in the nintedanib (orange) and placebo (purple) groups in the SENSCIS trial

In this *post-hoc* analysis, we explored FVC at baseline and decline in FVC in subjects in the SENSCIS trial in relation to a hypothetical reference population of healthy individuals (illustrated schematically in Fig. 1). The reference population comprised a group of hypothetical subjects that was matched 1:1 to subjects in the SENSCIS trial by age, sex, ethnicity and height (see Additional file 1: Appendix S1). FVC values in the reference subjects were determined using equations published by the European Respiratory Society Global Lung Function Initiative, which were based on data collected from over 70,000 healthy individuals aged 3–95 years from 26 countries [13]. Using the same reference equations, we estimated the “effective lung age” of subjects in the SENSCIS trial based on their FVC, sex, ethnicity and height (up to a limit of 95 years) and compared this to their real age (illustrated schematically in Fig. 1). The age of each subject as an integer was incorporated into the reference equations, which allows exact ages to be used without interpolation. As a limitation, this is only technically possible for subjects aged ≥ 25 years; thus, subjects aged < 25 years (n = 3) were excluded from our analyses.

In the nintedanib and placebo groups, we assessed FVC (mL) and effective lung age at baseline and at week 52, and changes in FVC (mL) at week 52. Missing FVC values at week 52 of the SENSCIS trial were imputed using predictions from the random slope and intercept model used in the primary analysis of the rate of decline in FVC [15]. Baseline FVC and changes in FVC (mL) at week 52 were compared in subgroups by mycophenolate use (yes, no), FVC % predicted (< 70, ≥ 70), time since onset of first non-Raynaud symptom (≤ 3, > 3 years), presence of cough (yes, no), and presence of dyspnoea (yes, no), all assessed at baseline. The presence of cough or dyspnoea was based on responses on the St George’s Respiratory Questionnaire (SGRQ) [19]: subjects who reported the symptom “most days a week”, “several days a week” or “a few days a month” (rather than “only with chest infection” or “not at all”) over the last month were considered to have that symptom. Differences in changes in FVC over 52 weeks between the subjects in the SENSCIS trial and the hypothetical healthy reference subjects were assessed using a paired *t*-test. P values < 0.05 were considered statistically significant but were not adjusted for multiplicity and should be interpreted descriptively.

## Results

### Subjects

A total of 576 subjects were treated in the SENSCIS trial (288 with nintedanib, 288 with placebo). The baseline characteristics of these subjects have been described [15]. Most subjects were female (75.2%) and white (67.2%). Mean (SD) age was 54.0 (12.2) years and median time since first non-Raynaud symptom was 3.4 years. Mean (SD) FVC was 72.5 (16.7) % predicted and DLco was 53.0 (15.1) % predicted; based on their responses to the SGRQ, 80.1% of subjects had cough and 70.0% had dyspnoea. Almost half (48.4%) of the subjects had been taking a stable dose of mycophenolate for ≥ 6 months.

### FVC at baseline

At baseline, mean (SD) FVC was 2460 (737) mL in the nintedanib group compared with 3403 (787) mL in the hypothetical healthy reference group. Mean (SD) FVC was 2544 (817) mL in the placebo group compared with 3516 (887) mL in the hypothetical healthy reference group. Baseline FVC was lower in subjects in the SENSCIS trial than in the reference subjects across subgroups based on mycophenolate use, FVC % predicted, time since first non-Raynaud symptom, and presence of cough or dyspnoea (Additional file 2: Table S1).

### Change in FVC from baseline to week 52

**Fig. 2:**
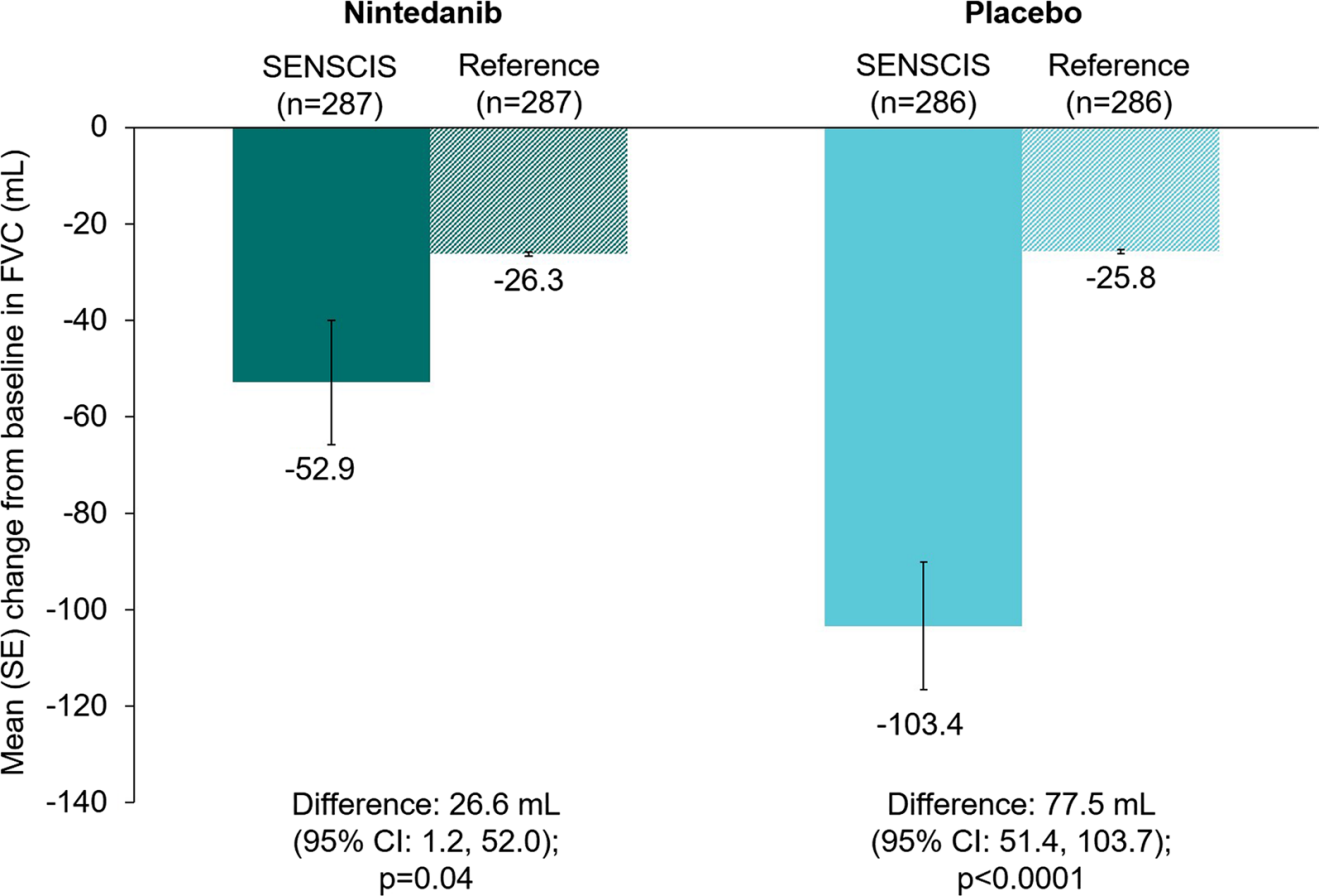
Change from baseline in FVC (mL) at week 52 in subjects in the SENSCIS trial versus matched hypothetical healthy references. Reference subjects were matched to the SENSCIS subjects for age, sex, ethnicity and height

The mean (SE) change in FVC at week 52 in the SENSCIS trial was − 52.9 (12.9) mL in the nintedanib group and − 103.4 (13.3) mL in the placebo group. The mean (SE) changes in FVC at week 52, i.e., age-related loss of lung function, in the hypothetical healthy references matched to the nintedanib and placebo groups, respectively, were − 26.3 (0.5) mL and − 25.8 (0.5) mL. The difference in the change in FVC at week 52 between the nintedanib group and the matched hypothetical healthy references was 26.6 mL (95% CI: 1.2, 52.0; p = 0.04). The difference in the change in FVC at week 52 between the placebo group and the matched hypothetical healthy references was 77.5 mL (95% CI: 51.4, 103.7; p < 0.0001) (Fig. 2).

**Fig. 3:**
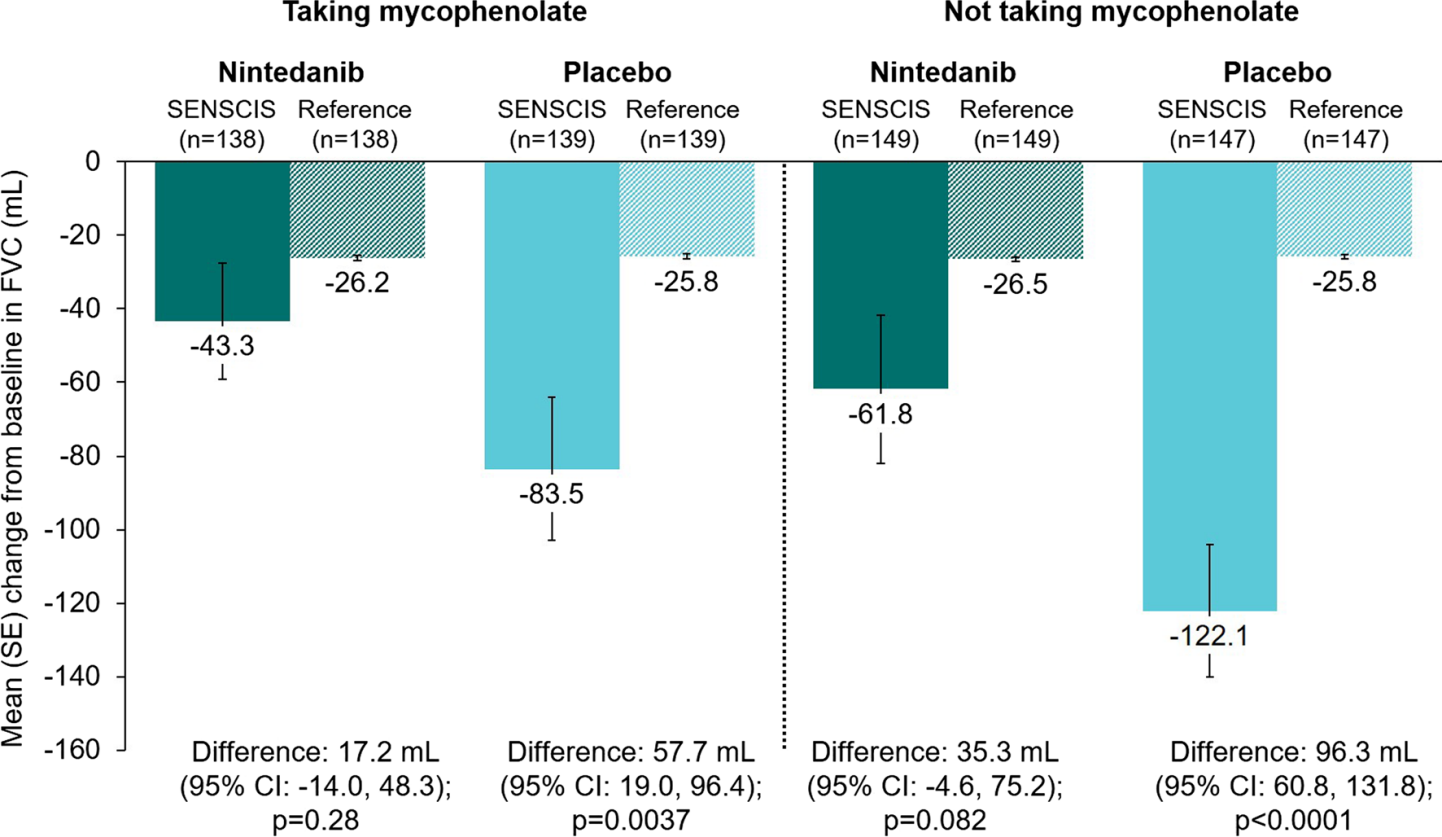
Change from baseline in FVC (mL) at week 52 in subjects in the SENSCIS trial versus matched hypothetical healthy references in subgroups by mycophenolate use at baseline. Reference subjects were matched to the SENSCIS subjects for age, sex, ethnicity and height

**Fig. 4:**
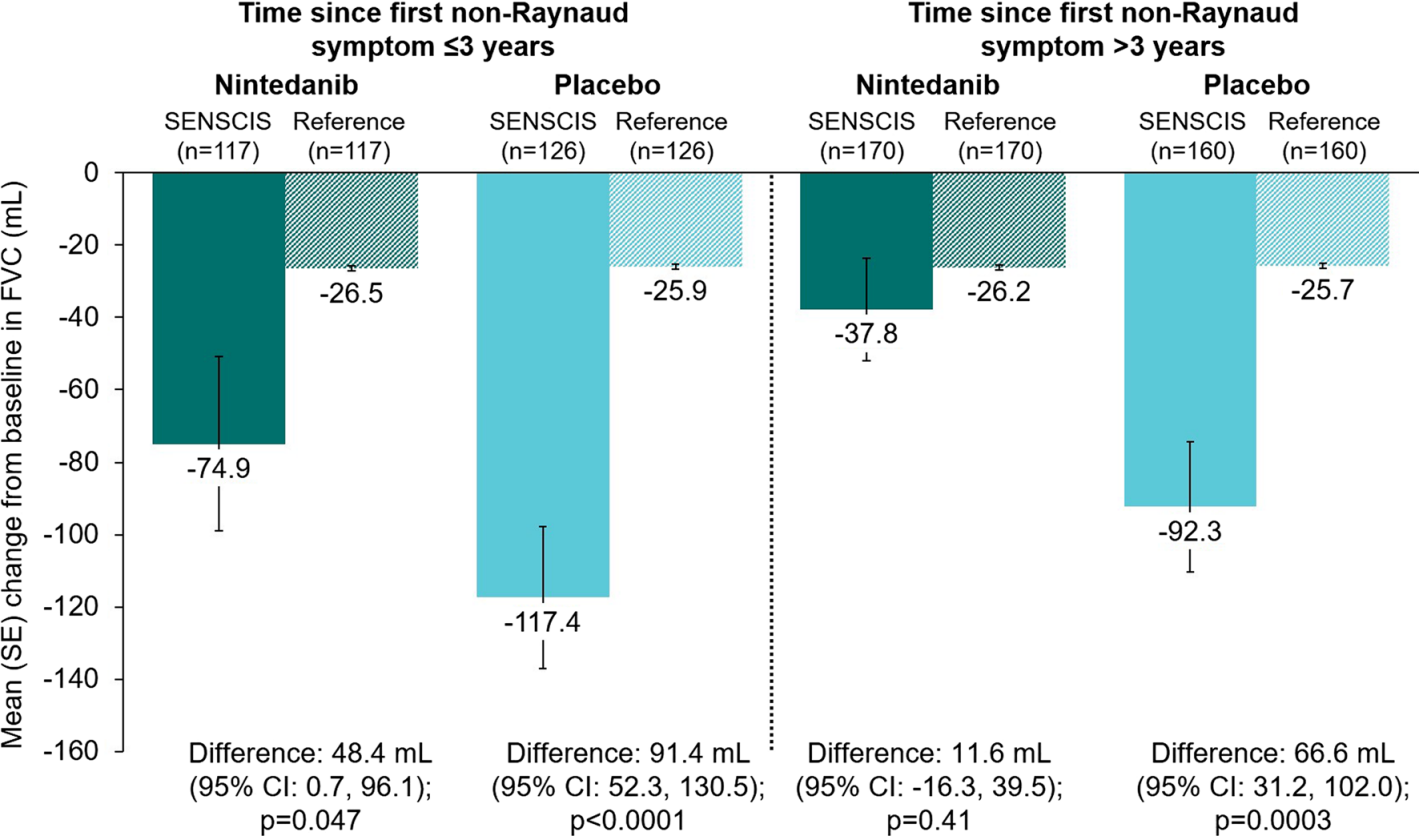
Change from baseline in FVC (mL) at week 52 in subjects in the SENSCIS trial versus matched hypothetical healthy references in subgroups by time since first non-Raynaud symptom. Reference subjects were matched to the SENSCIS subjects for age, sex, ethnicity and height

**Fig. 5:**
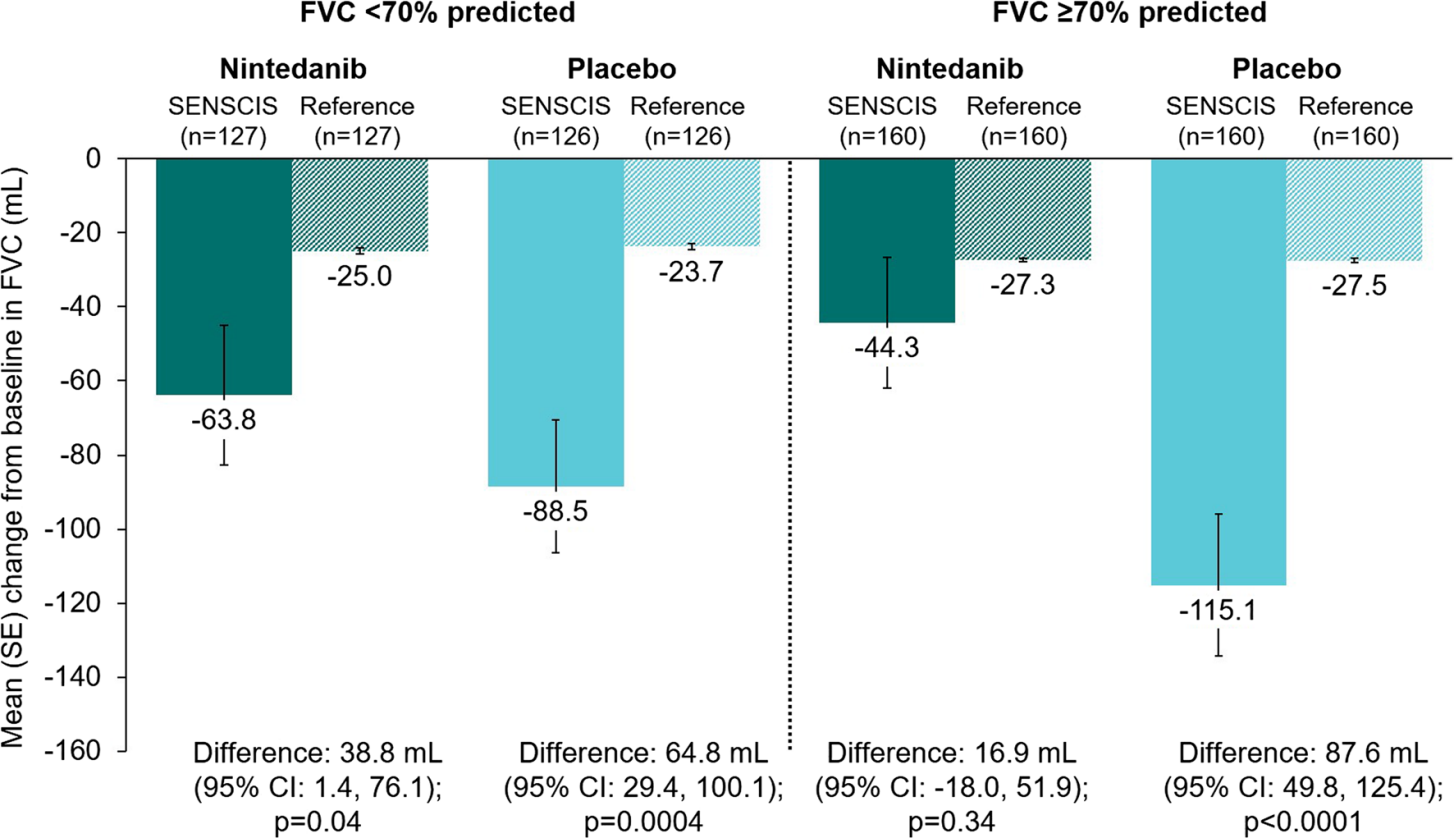
Change from baseline in FVC (mL) at week 52 in subjects in the SENSCIS trial versus matched hypothetical healthy references in subgroups by FVC % predicted at baseline. Reference subjects were matched to the SENSCIS subjects for age, sex, ethnicity and height

**Fig. 6:**
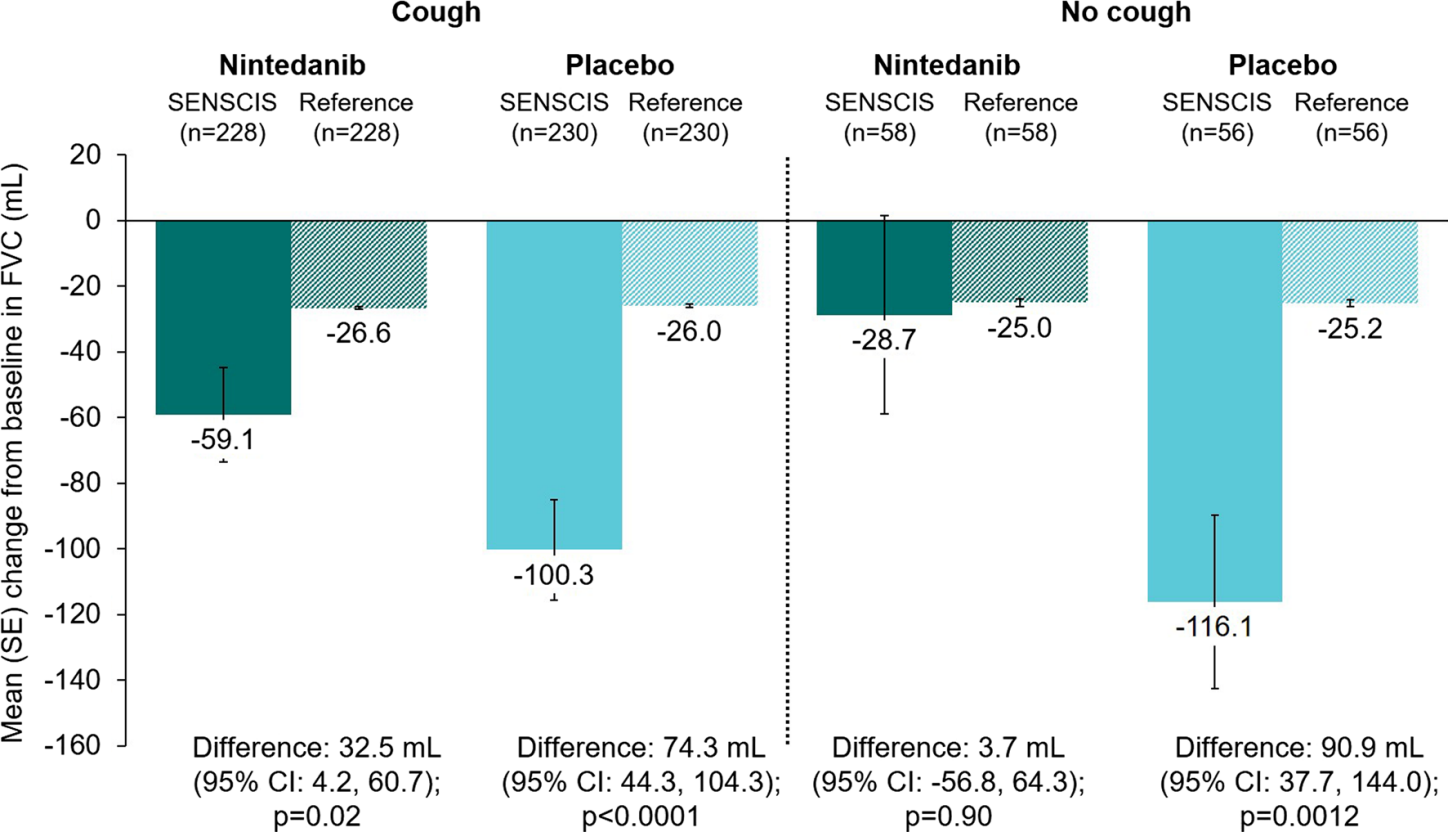
Change from baseline in FVC (mL) at week 52 in subjects in the SENSCIS trial versus matched healthy reference subjects in subgroups by cough at baseline. Reference subjects were matched to the SENSCIS subjects for age, sex, ethnicity and height

**Fig. 7:**
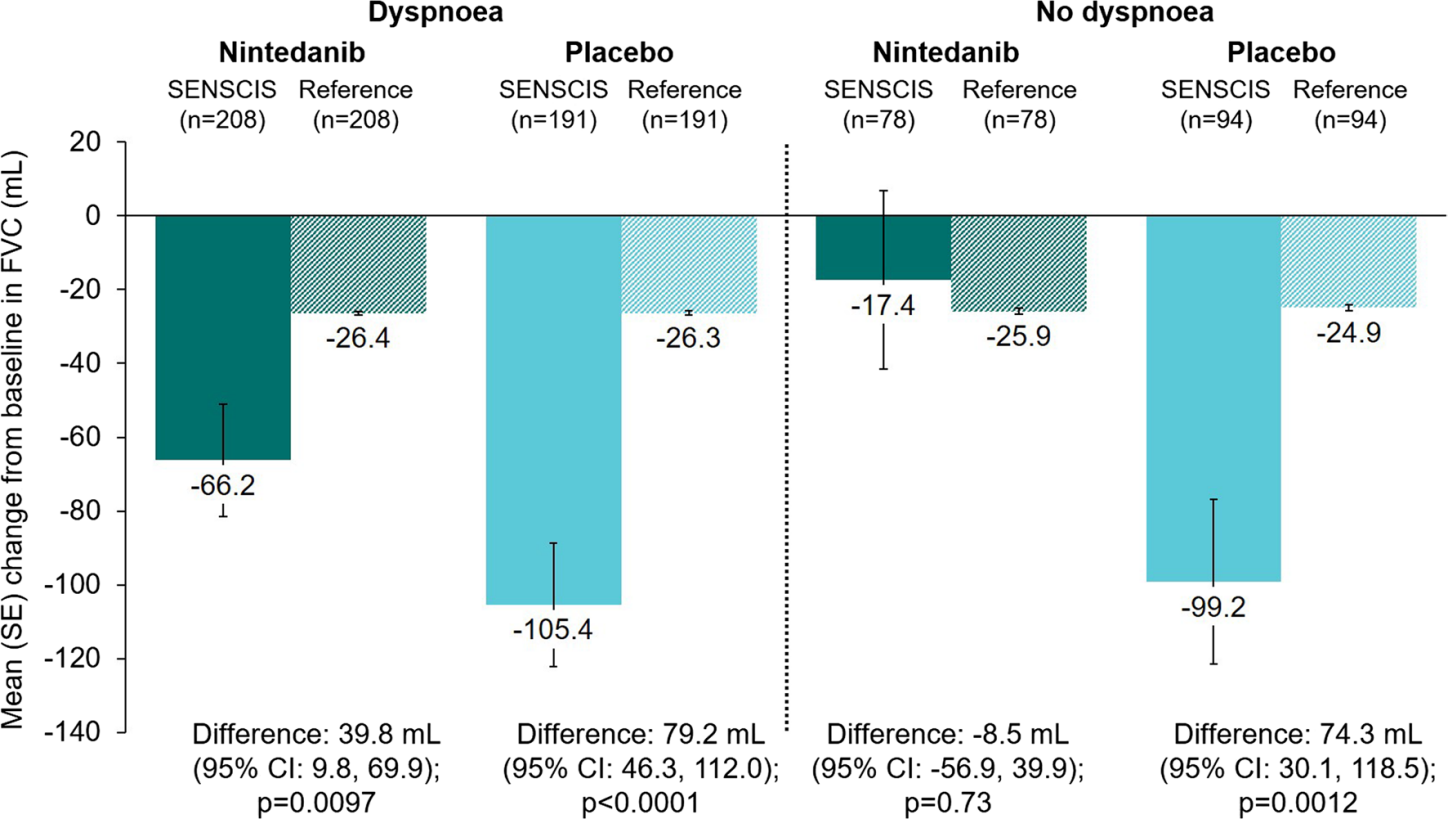
Change from baseline in FVC (mL) at week 52 in subjects in the SENSCIS trial versus matched hypothetical healthy references in subgroups by dyspnoea at baseline. Reference subjects were matched to the SENSCIS subjects for age, sex, ethnicity and height

There was no statistically significant difference in the change in FVC at week 52 between the nintedanib group and the hypothetical healthy references in the subgroups who did or did not use mycophenolate, or in the subgroups who had FVC ≥ 70% predicted, > 3 years since first non-Raynaud symptom, absence of cough, or absence of dyspnoea (Figs. 3, 4, 5, 6, 7). There were statistically significant differences in the change in FVC at week 52 between the placebo group and the hypothetical healthy references in each of the subgroups analysed (Figs. 3, 4, 5, 6, 7).

### Effective lung age

At baseline, mean (SD) effective lung age was 83.1 (14.4) years in the nintedanib group and 82.9 (14.8) years in the placebo group. The mean (SD) difference between effective lung age and real age at baseline was 28.4 (17.7) and 29.3 (18.5) years in these groups, respectively. In the nintedanib and placebo groups, respectively, 71.4% and 72.4% of subjects had a difference between effective lung age and real lung age of > 20 years. At baseline, median (Q1, Q3) effective lung age was 88.4 (74.6, 95.0) years in the nintedanib group and 88.5 (74.7, 95.0) years in the placebo group. At week 52, median (Q1, Q3) effective lung age was 91.0 (75.2, 95.0) years in the nintedanib group and 95.0 (75.9, 95.0) years in the placebo group.

## Discussion

The SENSCIS trial was conducted in a broad population of patients with SSc and at least 10% of the lungs affected by fibrotic ILD on an HRCT scan. The primary finding was that nintedanib was estimated to reduce the rate of decline in FVC (mL/year) over 52 weeks by an average of 44% compared with placebo [15]. To help put this finding into a meaningful context, we compared FVC at baseline and decline in FVC over 52 weeks in the SENSCIS trial with values in hypothetical subjects without lung disease, matched 1:1 for age, sex, ethnicity and height, estimated using reference equations [13]. To our knowledge, this is the first study to compare deterioration in lung function in patients with SSc-ILD with the decline that would be expected due to the natural ageing process.

At baseline, subjects in the SENSCIS trial had marked impairment in FVC compared with the matched reference population, with an FVC (mL) over 25% (about 950 mL) lower than would be expected in individuals without lung disease. This indicates that although recent progression of ILD was not an inclusion criterion for the SENSCIS trial, the subjects enrolled in the trial had experienced substantial decline in lung function. Given the average duration of SSc (time since first non-Raynaud symptom) in the subjects in the SENSCIS trial was about 3.4 years, this suggests a loss of lung function of about 280 mL/year since the onset of SSc, if one assumes a linear decline. However, previous studies suggest that that the rate of loss of lung function would likely be greatest in the earliest stages of the disease, particularly in the first 12 months after onset of symptoms [5, 6].

Over 52 weeks, the average decline in FVC in the placebo group of the SENSCIS trial was four-fold (78 mL) greater than in the hypothetical healthy references. This suggests that although evidence of recent ILD progression was not an inclusion criterion, on average, the patients enrolled in the SENSCIS trial experienced marked worsening of lung function over the following year. This finding is supported by real-world data from a recent study of 826 subjects with SSc-ILD in the EUSTAR database, in which 27% showed progression of ILD (defined as an FVC decline of > 10% predicted) over the first 12 months [3]. Among the 535 subjects who had ≥ 3 FVC measurements over a mean follow-up period of 5 years, 23% to 27% of subjects had progression within a given 12-month period, but periods of progression were rarely consecutive [3].

Subjects in the nintedanib group of the SENSCIS trial had an average decline in FVC over 52 weeks that was two-fold (27 mL) greater than in the hypothetical reference subjects. In subgroups with slower progression of SSc-ILD, such as those with > 3 years since first non-Raynaud symptom, FVC > 70% predicted, no cough, or no dyspnoea at baseline, the decline in FVC in subjects treated with nintedanib was close to the decline that would be expected in individuals without lung disease.

In both the nintedanib and placebo groups, the difference in the decline in FVC between subjects in the SENSCIS trial and the hypothetical healthy references was numerically lower in those taking mycophenolate at baseline. These data are in line with studies suggesting an improvement in FVC in patients taking mycophenolate [20, 21]; however, it should be remembered that the subjects taking mycophenolate were not randomized to receive mycophenolate but had been taking a stable dose of mycophenolate for ≥ 6 months prior to randomization. This lack of randomisation and potential biases preclude causal statements being made based on these data on the role of mycophenolate in reducing decline in FVC. Previous analyses of the SENSCIS trial indicated that nintedanib reduced the rate of decline in FVC both in subjects who were and were not taking mycophenolate at baseline, with no heterogeneity detected in its relative treatment between these subgroups [18, 22].

Calculating the “effective age” of organs and the body, and measuring risk and rate advancement periods (i.e., the impact of a risk factor on the timing of disease occurrence), can be useful tools for estimating and communicating health risks [23, 24]. At baseline of the SENSCIS trial, subjects with SSc-ILD had an effective lung age that was much higher than their real age (mean difference: 28.8 years). To our knowledge, this is the first time that effective lung age has been estimated in subjects with ILD. Over 52 weeks, the increase in effective lung age was numerically lower in subjects who received nintedanib than placebo. A reliable estimate of the difference in the increase in effective lung age over 52 weeks between the nintedanib and placebo groups could not be obtained, as so many patients were at the upper limit of effective lung age (95 years) at baseline and so could not experience an increase over 52 weeks.

Strengths of our analyses include the large and heterogeneous group of subjects enrolled in the SENSCIS trial, the standardised procedure for measuring FVC, and the use of reference equations for prediction of FVC that were derived from a large and diverse group of individuals with respect to age, ethnicity and geography. Limitations include the comparison of data from the SENSCIS trial with values from a hypothetical group of healthy references rather than real healthy subjects or subjects with systemic sclerosis but no ILD. Although the reference subjects were matched to the subjects in the SENSCIS trial by age, height, sex and ethnicity, other characteristics that may influence lung function, such as comorbidities, were not accounted for. Uncertainty in the predictions from the reference equations was not taken into account, but would likely have been small given the age of the subjects [13, 25].

## Conclusions

In conclusion, these analyses comparing baseline FVC and decline in FVC in the SENSCIS trial with a hypothetical group of matched healthy individuals show that the subjects in the SENSCIS trial, who were enrolled based on an extent of fibrotic ILD on HRCT of ≥ 10% but with no requirement for respiratory symptoms, had markedly impaired lung function at baseline and experienced further deterioration over 52 weeks. These analyses help to put the results of the SENSCIS trial into context and highlight the clinical relevance of the slowing of FVC decline provided by nintedanib.

## Supplementary Information

**Additional file 1: Appendix S1.** Calculation of FVC (mL) in reference subjects.

**Additional file 2: Table S1.** Baseline FVC (mL) in subjects in the SENSCIS trial versus healthy reference subjects in subgroups by baseline characteristics.

## Data Availability

To ensure independent interpretation of clinical study results, Boehringer Ingelheim (BI) grants all external authors access to relevant material, including participant-level clinical study data, as needed by them to fulfil their role and obligations as authors under the ICMJE criteria. Clinical study documents and participant clinical study data are available to be shared on request after publication of the primary manuscript in a peer-reviewed journal, and if regulatory activities are complete and other criteria met as per the BI Policy on Transparency and Publication of Clinical Study Data (https://www.mystudywindow.com/msw/datasharing). Bona fide, qualified scientific and medical researchers are eligible to request access to the clinical study data with corresponding documentation describing the structure and content of the datasets. Upon approval, and governed by a Legal Agreement, data are shared in a secured data-access system for a period of 1 year, which may be extended upon request. Prior to providing access, clinical study documents and data will be examined, and, if necessary, redacted and de-identified, to protect the personal data of study participants and personnel, and to respect the boundaries of informed consent. Researchers should use https://vivli.org to request access to study data and visit https://www.mystudywindow.com/msw/datasharing for further information.

## Abbreviations

ATS: Anti-topoisomerase I antibody
DLco: Diffusing capacity of the lungs for carbon monoxide
FVC: Forced vital capacity
HRCT: High resolution computed tomography
ILD: Interstitial lung disease
SGRQ: St George’s Respiratory Questionnaire
SSc: Systemic sclerosis
