# Supplementary material for: Decline in forced vital capacity in subjects with systemic sclerosis-associated interstitial lung disease in the SENSCIS trial compared with healthy reference subjects
Source: Respir Res. 2022 Jul 5;23:178. doi: 10.1186/s12931-022-02095-6 (PMC9258095; doi:10.1186/s12931-022-02095-6)
Supplement: Supplementary file 1 — Additional file 1: Appendix S1. Calculation of FVC (mL) in reference subjects. [file 12931_2022_2095_MOESM1_ESM.docx]

**Additional File 1: Appendix S1**

**Calculation of FVC (mL) in reference subjects**

The calculation of FVC in the reference subjects was based on equations published by the European Respiratory Society Global Lung Function Initiative [1].

**Step 1**

MSPLINE = b_0_ + b_1_*(Age/100) + b_2_*(Age/100)^2^ + b_3_*(Age/100)^3^ + b_4_*(Age/100)^4^ + b_5_*(Age/100)^5^

with:

| **Males** | | | **Females** | | |
| --- | --- | --- | --- | --- | --- |
| Intercept | b_0_ | 0.3298 | Intercept | b_0_ | 0.0745 |
| (Age/100) | b_1_ | -1.1230 | (Age/100) | b_1_ | 0.6006 |
| (Age/100)^2^ | b_2_ | 2.8110 | (Age/100)^2^ | b_2_ | -1.0684 |
| (Age/100)^3^ | b_3_ | -5.4811 | (Age/100)^3^ | b_3_ | -1.1308 |
| (Age/100)^4^ | b_4_ | 3.5964 | (Age/100)^4^ | b_4_ | 0.9730 |
| (Age/100)^5^ | b_5_ | -0.5884 | (Age/100)^5^ | b_5_ | 0.0643 |

Age at informed consent was considered as age at baseline. Age at week 52 was derived as (age at baseline)+1.

**Step 2**

MSPLINE was plugged into equation for M:
M = exp(a_0_ + a_1_*ln(Height) + a_2_*ln(Age) + a_3_*Afr-Am + a_4_*NEAsia + a_5_*SEAsia + a_6_*Other + MSPLINE)

with:

| **Males** | | | **Females** | | |
| --- | --- | --- | --- | --- | --- |
| Intercept | a_0_ | -11.2281 | Intercept | a_0_ | -10.4030 |
| Height | a_1_ | 2.4135 | Height | a_1_ | 2.2633 |
| Age | a_2_ | 0.0865 | Age | a_2_ | 0.0234 |
| Afr-Am | a_3_ | -0.1684 | Afr-Am | a_3_ | -0.1555 |
| NE Asia | a_4_ | -0.0405 | NE Asia | a_4_ | -0.0262 |
| SE Asia | a_5_ | -0.1177 | SE Asia | a_5_ | -0.1516 |
| Other/mixed | a_6_ | -0.0825 | Other/mixed | a_6_ | -0.0833 |

Mapping of race categories provided by the European Respiratory Society Global Lung Function Initiative [1] to race and country information collected in the SENSCIS trial [2]:

| **Race category in** Global Lung Function Initiative equations [1] | **Race category in SENSCIS trial** | **Country in SENSCIS trial** |
| --- | --- | --- |
| Caucasian | White |  |
| African-American | Black or African-American |  |
| NE Asian | Not applicable/available |  |
| SE Asian | Asian | Malaysia, Thailand |
| Other | Missing or Asian | Not in Malaysia or Thailand |

**References**

1. Quanjer PH, Stanojevic S, Cole TJ, et al. Multi-ethnic reference values for spirometry for the 3-95-yr age range: the global lung function 2012 equations. Eur Respir J 2012;40:1324–1343.
2. Distler O, Highland KB, Gahlemann M, et al. Nintedanib for systemic sclerosis-associated interstitial lung disease. N Engl J Med 2019;380:2518–2528.
