# Supplementary material for: Decline in forced vital capacity in subjects with systemic sclerosis-associated interstitial lung disease in the SENSCIS trial compared with healthy reference subjects
Source: Respir Res. 2022 Jul 5;23:178. doi: 10.1186/s12931-022-02095-6 (PMC9258095; doi:10.1186/s12931-022-02095-6)
Supplement: Supplementary file 2 — Additional file 2: Table S1. Baseline FVC (mL) in subjects in the SENSCIS trial versus healthy reference subjects in subgroups by baseline characteristics. [file 12931_2022_2095_MOESM2_ESM.docx]

**Additional File 2: Table S1**

Baseline FVC (mL) in subjects in the SENSCIS trial versus healthy reference subjects in subgroups by baseline characteristics

|  | **Nintedanib** | | **Placebo** | |
| --- | --- | --- | --- | --- |
|  | **SENSCIS** | **Reference** | **SENSCIS** | **Reference** |
| Mycophenolate use |  |  |  |  |
| Yes | 2499 (726) | 3569 (754) | 2581 (816) | 3666 (897) |
| No | 2423 (748) | 3250 (789) | 2509 (820) | 3375 (856) |
| FVC % predicted |  |  |  |  |
| <70 | 2035 (504) | 3513 (788) | 2073 (591) | 3606 (914) |
| ≥70 | 2797 (719) | 3316 (778) | 2915 (779) | 3446 (860) |
| Time since first non-Raynaud symptom, years |  |  |  |  |
| ≤3 | 2512 (793) | 3467 (863) | 2587 (849) | 3488 (919) |
| >3 | 2424 (696) | 3360 (731) | 2510 (792) | 3539 (862) |
| Cough based on response to question in SGRQ* |  |  |  |  |
| Yes | 2445 (703) | 3438 (808) | 2539 (842) | 3544 (897) |
| No | 2511 (867) | 3233 (643) | 2565 (714) | 3402 (840) |
| Dyspnoea based on response to question in SGRQ* |  |  |  |  |
| Yes | 2396 (673) | 3402 (755) | 2535 (832) | 3566 (925) |
| No | 2623 (871) | 3384 (851) | 2570 (790) | 3411 (802) |

Data are mean (SD). In the nintedanib and placebo groups of SENSCIS, respectively, n=138 and n=139 were taking mycophenolate and n=149 and n=147 were not taking mycophenolate; n=127 and n=126 had FVC <70% predicted and n=160 and 160 had FVC ≥70% predicted; n=117 and n=126 had time since first non-Raynaud symptom ≤3 years and n=170 and n=160 had time since first non-Raynaud symptom >3 years; n=228 and n=230 had cough and n=58 and n=56 did not have cough at baseline; n=208 and n=191 had dyspnoea and n=78 and n=94 did not have dyspnoea. *Subjects who reported the symptom “most days a week”, “several days a week” or “a few days a month” (rather than “only with chest infection” or “not at all”) over the last month were considered to have that symptom.
